# Astrocyte adaptation in Alzheimer’s disease: a focus on astrocytic P2X_7_R

**DOI:** 10.1042/EBC20220079

**Published:** 2023-03-03

**Authors:** Paula Beltran-Lobo, Matthew J. Reid, Maria Jimenez-Sanchez, Alexei Verkhratsky, Beatriz G. Perez-Nievas, Wendy Noble

**Affiliations:** 1 Department of Basic and Clinical Neuroscience, King’s College London, Institute of Psychiatry, Psychology and Neuroscience, 5 Cutcombe Road, London, SE5 9RX, U.K.; 2 Faculty of Biology, Medicine and Health, The University of Manchester, Manchester, U.K.; 3 Achucarro Center for Neuroscience, IKERBASQUE, 48011 Bilbao, Spain; 4 Department of Forensic Analytical Toxicology, School of Forensic Medicine, China Medical University, Shenyang, China; 5 Department of Stem Cell Biology, State Research Institute Centre for Innovative Medicine, LT-01102, Vilnius, Lithuania

**Keywords:** Alzheimers disease, astrocytes, p2x7, synapses, tau proteins

## Abstract

Astrocytes are key homeostatic and defensive cells of the central nervous system (CNS). They undertake numerous functions during development and in adulthood to support and protect the brain through finely regulated communication with other cellular elements of the nervous tissue. In Alzheimer’s disease (AD), astrocytes undergo heterogeneous morphological, molecular and functional alterations represented by reactive remodelling, asthenia and loss of function. Reactive astrocytes closely associate with amyloid β (Aβ) plaques and neurofibrillary tangles in advanced AD. The specific contribution of astrocytes to AD could potentially evolve along the disease process and includes alterations in their signalling, interactions with pathological protein aggregates, metabolic and synaptic impairments. In this review, we focus on the purinergic receptor, P2X_7_R, and discuss the evidence that P2X_7_R activation contributes to altered astrocyte functions in AD. Expression of P2X_7_R is increased in AD brain relative to non-demented controls, and animal studies have shown that P2X_7_R antagonism improves cognitive and synaptic impairments in models of amyloidosis and tauopathy. While P2X_7_R activation can induce inflammatory signalling pathways, particularly in microglia, we focus here specifically on the contributions of astrocytic P2X_7_R to synaptic changes and protein aggregate clearance in AD, highlighting cell-specific roles of this purinoceptor activation that could be targeted to slow disease progression.

## Introduction

Neuropathological changes in Alzheimer’s disease (AD) include the progressive deposition of senile plaques and neurofibrillary tangles (NFTs), alongside extensive and complex glial alterations, vascular changes, synapse and neuron loss, leading to cognitive impairment and dementia [1].

Astrocytes are a subpopulation of glial cells derived from neuroepithelial progenitors that account for 20–40% of total glial cells in humans, depending on the brain region [2,3]. Single-cell transcriptomics revealed considerable molecular heterogeneity among astrocyte populations in rodent brain [4] as well as a complex stratified architecture across cerebral layers [5] that is likely more diverse in human brain, which in addition contain several forms of astrocytes absent in other mammals [6].

Astrocytes perform critical functions in the developing and adult CNS [7]. For example, during development, astrocytes remodel neuronal circuits, participating in the formation and pruning of synapses [8,9]. Astrocytes are functionally integrated with synapses, with all astrocytic compartments found to abut synapses in adult mouse hippocampus [10]. A particularly high density of pre-synaptic terminals and/or dendritic spines contact astrocytic branches and leaflets [10,11] which may result from these being amongst the most dynamic of astrocytic structures in response to neuronal signals [10]. Astrocytes, together with other cellular and non-cellular elements, form multipartite synapses that regulate various aspects of synaptic function [12]. Astrocytes control synaptogenesis, synaptic maturation, synaptic maintenance and synaptic extinction through the release of multiple specific regulatory molecules including thrombospondins, hevins, glypicans, norrin and many more [13]. Astrocytes modulate synaptic activity by several mechanisms of which K^+^ buffering, glutamate clearance by astrocyte specific excitatory amino acid transporter (EAAT)1/2 receptors and supplying glutamine by the glutamate-(GABA)-glutamine shuttle are of particular relevance [14]. Astrocytes release small molecules such as adenosine-triphosphate (ATP) which is rapidly converted to adenosine by ectonucleotidases to supress excitatory transmission by acting on presynaptic adenosine A_1_ receptors (A_1_R) [15,16]. Astrocytes also participate in the maintenance of ion homeostasis allowing the rapid uptake of K^+^ from the extracellular space during neuronal activity [17]. Thus, astrocytes are actively engaged in the regulation of synaptic transmission, synaptic plasticity and maintenance of neuronal circuitry in the CNS, as previously reviewed [11].

In addition, astrocytic end-feet are a parenchymal component of the blood–brain barrier (BBB). They contribute to the regulation of blood flow in response to neuronal activity, along with perivascular neuronal terminals, endothelial cells and pericytes, in a process known as neurovascular coupling [18]. Astrocytes take up glucose, the main energy source of the brain, and store it as glycogen, or may supply it to neurons in the form of lactate [19,20], although this is debated. Furthermore, end-feet-specific expression of aquaporin-4 (AQP4) maintains the proper function of glymphatic system which facilitates the elimination of toxic solutes from the interstitial fluid [21].

### Astrocytes in AD

Brain injury associated with trauma, stroke, neuroinfection or immune attack triggers specific and stereotypical defensive responses of astrocytes known as reactive astrogliosis in which astrocytes proliferate, form a glial scar and promote the recruitment of immune cells. In neurodegenerative diseases including AD, astrocytes undergo morphological, molecular and functional changes commonly known as astrocytic reactivity [22]. In contrast to the *bona fide* stereotypical reactive astrogliosis that is triggered by brain lesions associated with a breach of the BBB, astrocytic reactivity in chronic neurological diseases is highly dynamic and heterogeneous [22].

Glial fibrillary acidic protein (GFAP) is a cytoskeletal protein in astrocytes that is increased to allow cytoskeletal rearrangement in response to many physiological and disease stimuli [22–25]. In human AD brain, astrocytes expressing different isoforms and/or truncated forms of GFAP cluster around Aβ plaques [26,27]. This is similar in mice where, without altering their spatial distribution, astrocytes extend their processes towards amyloid deposits [28]. Astrocytes with GFAP-immunoreactivity are also associated with ghost neurofibrillary tangles (NFTs) in advanced stages of AD [29], a feature believed to result from astrocyte processes having penetrated extracellular ghost tangles or tangle-bearing neurons in advanced AD and becoming separated from the soma [30–32]. Moreover, increased numbers of GFAP-immunoreactive astrocytes in the superior temporal sulcus are among the molecular features that distinguish between AD cases with dementia and those showing resilience to cognitive decline [33].

Levels of GFAP in brain tissue are high in prodromal AD [34] and an increase in the levels of GFAP in cerebrospinal fluid (CSF) and plasma of AD patients is also detected in early stages, that peaks upon symptom onset [35]. Recent work using specific positron electron tomography (PET) ligands demonstrated an association between Aβ, but not tau, burden and CSF GFAP levels [36]. These authors further showed that the strength of tau-PET signals is more closely associated with increases in CSF levels of a putative marker of astrocyte reactivity YKL-40 [37] that is expressed only in a subset of astrocytes [38] and also by several other cell types [39]. Others have reported that CSF YKL-40 levels are distinct from grey matter loss associated with phosphorylated tau [40].

However, characterisation of GFAP immunostaining is insufficient to make conclusions about functional changes in astrocytes. Indeed, astrocytes show considerable transcriptomic heterogeneity in the diseased brain [41], even amongst cases with the same diagnosis [42]. Recent multi-transcriptomic analysis of human astrocytes in AD relative to control brain revealed increases in genes related to specialized astrocyte-neuron contacts at perisynaptic astrocyte leaflets that influence the function of adjacent synapses, alongside downregulation of endolysosomal and mitochondrial genes in astrocytes, that for mitochondrial genes were found to decline as disease severity increased [42]. The decrease in endolysosomal and mitochondria-related genes, but not the upregulation of synapse-related genes, was mirrored in transgenic APP/PS1 mice [42].

**Figure 1:**
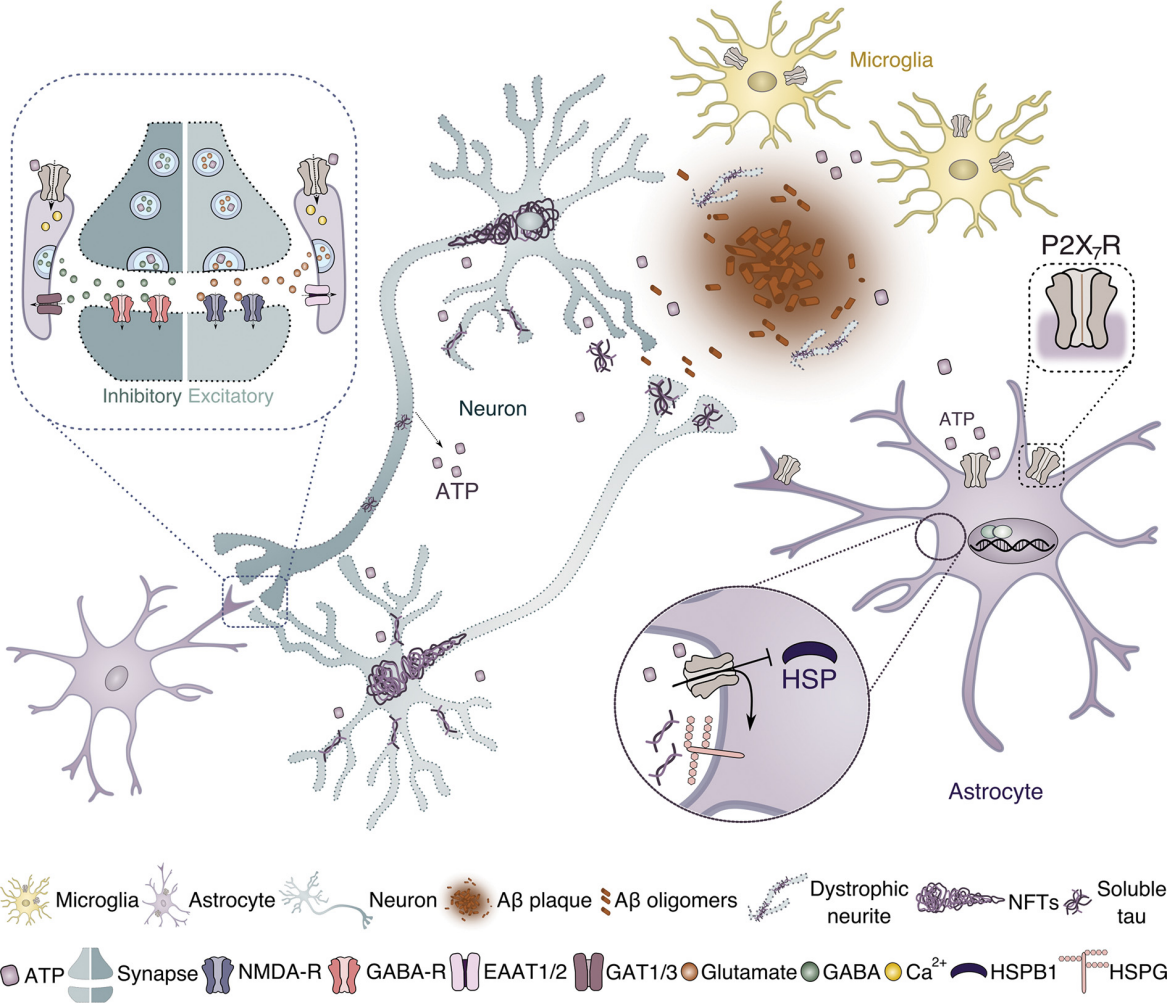
Potential roles of astrocytic P2X_7_R in synaptic function and protein clearance in AD In AD brain, high levels of ATP could activate P2X_7_R in astrocytes and contribute to defects in neurotransmission. The opening of P2X_7_R channels allows calcium influx which modulates the release of glutamate to the synaptic cleft, where it could bind to NMDA-Rs at the post-synapse. P2X_7_R could also participate in the regulation of inhibitory synapses, by modulating the release of GABA from astrocytes. In addition to their intimate association with synapses, astrocytes also play important roles in the maintenance of protein homeostasis through the internalisation and degradation of Aß and tau aggregates. In AD, astrocytic P2X_7_R could alter protein clearance pathways via HSPB1-mediated autophagy or the regulation of HSPG expression, which might influence astrocyte uptake and clearance of tau species.

Astrocytes, like microglia, possess a myriad of cell surface receptors through which they recognise a variety of stimuli [43]. These include molecules released upon cell damage including adenosine triphosphate (ATP) which is rapidly converted to adenosine by ectonucleotidases, heat-shock proteins, and disease-specific pathological species of Aβ and tau [44–47]. The binding of these pathogenic molecules to astrocytic receptors is a key step for the initiation of signal transduction cascades that increase the transcription of target genes [48,49]. Below, we discuss how sensing through one of these receptors, the purinergic P2X_7_ receptor, may contribute to alterations in synaptic and endolysosome-related functions of astrocytes in AD (Figure 1).

## Purinergic receptors

The purinoreceptor family includes highly conserved receptors that bind adenine (P0 receptors), adenosine (P1 receptors) and ADP/ATP (P2 receptors) [50–53]. P2 receptors are further classified into G-protein coupled (P2Y) and ion-gated (P2X) receptors [54]. To date, seven P2X (PX_1-7_R) and eight P2Y (P2Y_1_R, P2Y_2_R, P2Y_4_R, P2Y_6_R, P2Y_11-14_R) subtypes have been identified, including P2Y receptors with the ability to sense pyrimidines (e.g., uridine diphosphate [UDP], UDP-glucose, UDP-galactose) in addition to purines [55].

## P2X_7_R

The P2X_7_R protein consists of a short intracellular N-terminal domain, two transmembrane α-helixes, an extracellular loop enriched in N-glycosylation sites and a long cytoplasmatic C-terminal domain [56,57]. P2X_7_R is typically found in a resting/closed or apo-state conformation, with a narrow cavity through which ATP must access to reach the active binding pocket. When ATP binds to P2X_7_R, conformational rearrangements lead to the opening of an ion-permeable channel that allows the influx (i.e., Na^+^, Ca^2+^) and efflux (i.e., K^+^) of small cations, and upon channel dilatation into a larger pore, the entry of large hydrophilic molecules at a slower rate [58]. In contrast to other P2X subtypes, P2X_7_R does not undergo desensitisation after activation of the receptor due to permanent stabilization provided by a palmitoylated cysteine rich region in the cytoplasmatic domain [59]. This feature of P2X_7_R activation dynamics likely contributes to the hyperpolarised astrocyte membrane potential that is important for astrocyte physiology and functions [60].

The human P2X_7_R gene is highly polymorphic with more than 150 non-synonymous SNPs, the majority of which lead to amino acid substitutions in the extracellular loop or the cytoplasmic C-terminal tail [61], affecting agonist binding affinity [62], trafficking to membranes [63], ion channel activity [64] and permeability of the pore [65]. In humans, there are seven splice variants of P2X_7_R, two of which are predominantly expressed in the CNS and immune tissues including the full-length (P2X_7_R A) and a C-terminally truncated form with an early stop codon (P2X_7_R B) [66,67]. The latter gives rise to a receptor deficient in the formation of a large permeable pore that retains ion channel properties [67].

Opening of the P2X_7_R channel is stimulated by ATP concentrations in the higher micromolar to millimolar range, in contrast to the lower ATP concentrations required for opening of other P2X family members, with ATP^4−^ being a true agonist [68]. ATP can be sensed by P2X_7_Rs in rodent or human microglia which show the highest levels of P2X_7_R expression in the brain [69–72] and oligodendrocytes [73,74]. Whilst the neuronal localization of P2X_7_R remains controversial [75,76], P2X_7_R expression in astrocytes [71,72,77–80] has been confirmed by the presence of agonist-induced currents and Ca^2+^ responses [78,81–86]. Activation of P2X_7_R in astrocytes triggers various cellular signals including those that stimulate nitrous oxide and superoxide radicals production [87,88], Akt and mitogen-activated protein kinase (MAPK) signalling [89–91], secretion of cytokines and other mediators of inflammation [88,89,92–94], as well as the release of gliotransmitters [81,95,96].

### P2X_7_R in AD

While there is limited evidence that single nucleotide polymorphisms that alter *P2X_7_R* activity influence the risk of AD [97], converging studies show enhanced levels of *P2X_7_R* mRNA and P2X_7_R protein in AD post-mortem brains in comparison with non-demented controls suggesting an involvement of this purinoreceptor in AD [71,98,99]. This increase is similarly observed in several transgenic mouse models of familial AD that overexpress mutant forms of *APP* including Tg2576 [80], APP/PS1 [100] and J20 [99] mice as well as in tauopathy models carrying mutations in *MAPT* [63]. Higher levels of P2X_7_R were observed at 12 months relative to 3-month-old APP/PS1 mice suggesting that P2X_7_R expression increases with disease progression [100]. P2X_7_R protein increases are particularly apparent surrounding Aβ plaques in AD brain [71,99,101], and this plaque-associated up-regulation is recapitulated in transgenic rodent models of amyloidogenesis [80,99,100]. In addition to changes in P2X_7_R expression, pharmacological antagonism or genetic deletion of P2X_7_R protects against disease in mouse models harbouring Aß [62,92] or tau pathology [63,91,93] indicating that P2X7R-mediated functions contribute to the disease process.

### P2X_7_R contributions to astrocyte driven synaptic changes in AD

Astrocytes are implicated in the deterioration of synaptic transmission in AD, affecting both excitatory (glutamatergic) and inhibitory γ-aminobutyric acid (GABA)-ergic synapses [14]. Aβ induces calcium dysregulation in astrocytes which can affect their ability to modulate neurotransmission [102,103]. For example, astrocytes can induce neuronal hyperactivity through the transient receptor potential cation channel, subfamily A, member 1 (TRPA1) in response to Aβ, which potentiates the release of excitatory glutamate in APP/PS1 mice [104]. Reports describing an increase in the release of the N-methyl-D-aspartate receptor (NMDA-R) co-factor D-serine from these astrocytes are now questioned, since astrocytes do not produce D-serine but rather L-serine which can be shuttled to neurons to drive neuronal production of D-serine [105]. Compromised glucose metabolism is observed in prodromal stages of AD which correlates with disease progression [14,106]. Accordingly, reduced aerobic glycolysis is also observed in prodromal AD [107], one consequence of which is decreased L-serine synthesis by astrocytes [108]. This disrupts NMDA-R-mediated synaptic plasticity and cognitive function in AD mice, which can be recovered upon dietary L-serine supplementation [109].

In transgenic mice expressing AD-causing mutant forms of *APP* and *PSEN1* (APP/PS1), astrocytes surrounding Aβ plaques have lower levels of EAAT2, which leads to an extra-synaptic accumulation of glutamate, neuronal hyperactivity [110] potentially mediated by neuronal NMDA receptors [111], and possibly neurotoxicity. However, whether or not this is true in human disease is uncertain since analysis of postmortem brain from AD cases with significant amyloid and tau pathology showed higher levels of astrocytic EAAT2 in comparison with non-demented cases carrying AD pathology, pointing towards a mechanism of astrocytic resilience against neuropathological changes in AD [112]. Astrocytic P2X_7_R could be activated by ATP, or potentially indirectly, by Aβ [113], in the vicinity of senile plaques to contribute to excess glutamate levels. Stimulation of P2X_7_R in hippocampal, spinal cord and substantia gelatinosa astrocytes using the potent broad agonist 2′(3′)-O-(4-benzoylbenzoyl)adenosine 5′-triphosphate (BzATP) induces glutamate release and stimulation of neighbouring NMDA-Rs through a Ca^2+^-dependent mechanism [114,115]. The opening of P2X_7_R pores could also mobilise other transmitter-containing vesicles following Ca^2+^ entry, but the precise molecular pathways that mediate these events remain obscure [75]. Astrocytes can also influence neuronal inhibition by increasing GABA release at the synaptic cleft in mice expressing five familial mutations in *APP* and *PSEN1* (5xFAD mice, [116]), and some GABA release from astrocytes is regulated by P2X_7_Rs, at least in stratum radiatum astrocytes proximal to interneurons in the hippocampus [117].

### P2X_7_R and astrocytic protein clearance pathways – implications for AD

Recent analysis showed that endolysosomal pathway components, fundamental for the uptake, processing, degradation and disposal of proteins and cellular debris, are down-regulated in AD astrocytes [42]. Indeed astrocytes, in addition to microglia, play central roles in the clearance of protein aggregates and other debris in degenerating AD brain [118]. By surrounding Aβ plaques, glia erect a physical and functional barrier to isolate and potentially clear proteinaceous aggregates from the affected neuropil [119].

Aß oligomers are observed within astrocytes in post-mortem AD brain [120] and mature healthy astrocytes engulf and degrade Aβ species *in vitro* and *ex vivo* [121,122]. Inhibition of reactive astrogliosis either increases [123] or reduces [124] levels of Aβ in APP/PS1 mice. The astrocyte-mediated internalisation of Aβ occurs in a ApoE-dependent manner, since *ApoE* deficient astrocytes are not capable of removing amyloid [125], with efficient Aβ uptake and clearance from astrocytes dependent on transcription factor EB (TFEB)-mediated lysosomal degradation [126]. Similarly, there is evidence that the Aβ sensor low density lipoprotein receptor-related protein 1 (LRP1) is critical for astrocytic uptake and degradation of Aß [47]. Astrocytes can also upregulate the expression of extracellular proteolytic enzymes that target Aβ including insulin degrading enzyme [127], released via an unconventional autophagy-dependent secretory pathway, and endothelin-converting enzyme-2 [128], they are efficient in autophagy and can potentially limit the accumulation of Aβ species in AD [118].

Astrocytes can also internalize modified forms of tau protein. In a tauopathy mouse model in which tau was expressed specifically in entorhinal cortex neurons, tau aggregates that spread trans-synaptically to the dentate gyrus were detected in astrocytes [129]. These data indicate that astrocytes internalize and may contribute to tau propagation. Indeed, extracellular forms of fibrillar tau are taken up by astrocytes [46], including at synapses for redirection into lysosomal degradation pathways to regulate tau spread [130]. Data also implicates heparin sulphate proteoglycans (HSPGs) and LRP1 in tau uptake by astrocytes, with the efficiency of the uptake varying depending on disease-associated tau modifications [131–134].

While the direct contributions of astrocytic P2X_7_R to these processes have not been investigated in detail, several independent studies demonstrated that pharmacological blockade or genetic deletion of P2X_7_R is beneficial in mouse models of AD, reporting reduced amyloid plaque number and abundance of soluble Aβ species in mouse models of amyloidosis [71,135]. In tauopathy mouse models, decreases in tau phosphorylation at certain epitopes [72,101] or a reduction in the abundance of misfolded tau forms [136] were reported. Although some alterations in microglial morphology and functions including phagocytosis, migration and cytokine release were observed upon P2X_7_R inhibition [72,101], no consistent changes were detected between the different mouse models [71,136]. No alterations in protein degradation pathways were described although P2X_7_R induction in microglia is known to impair lysosomal function, increasing levels of the autophagosome membrane-associated form of microtubule-associated protein 1 light chain 3 (LC3)-II in a Ca^2+^-dependent manner, up-regulating the formation of autophagosomes and autophagolysosomes, and increasing the release of autophagosomes [137,138]. We suggest that further exploration of the potential contribution of astrocytic P2X_7_R to these processes is warranted since P2X_7_R activation also regulates autophagy in astrocytes [139,140]. Astrocytes are damaged in status epilepticus, and they form vacuoles containing lysosome-associated membrane protein (LAMP)-1 [141]. P2X_7_R antagonism was found to decrease astrocyte damage in the molecular layer of the dentate gyrus and frontoparietal cortex under these conditions [142] which could be caused by a prolonged induction of the molecular chaperone small heat-shock protein (HSP)B1, a HSP that facilitates the folding and removal of aberrant proteins, and, in turn, promotes astroglial autophagy [139]. Others have shown similar effects in P2X_7_R knockout mice, where P2X_7_R signalling to focal adhesion kinase (FAK) was found to regulate HSPB25 and fine tune autophagy [140]. Since, as we discuss above, astrocytes efficiently internalise Aβ and modified forms of tau in disease, these data may suggest that P2X_7_R-induced regulation of autophagy in astrocytes is important for limiting proteinaceous spread in AD and tauopathies. Finally, tau internalization and release may be mediated by HSPGs [143,144]. P2X_7_R also regulates HSPG expression and localization, at least in the cornea [145], and we suggest that exploration of the potential for P2X_7_R signalling to similarly affect HSPGs in astrocytes and alter tau clearance is warranted.

In summary, we provide an overview of synapse-related and protein clearance functions of astrocytes that can be modulated by P2X_7_R signalling in AD. P2X_7_R has gained much attention in recent years as a possible therapeutic target. Genetic deletion of P2X_7_R in APP/PS1 mice improved long-term synaptic plasticity, spatial learning and memory dysfunction relative to wild-type littermates [71], and P2X_7_R antagonism in mouse models of tauopathy harbouring the frontotemporal dementia (FTD)-causing *MAPT* mutations G272V and/or P301S ameliorates cognitive and behavioural deficits as well as synaptic dysfunction [72,101,136]. We suggest that further exploration of P2X_7_R-driven effects on biological processes linked with astrocyte contributions to AD may uncover novel targets for therapeutic intervention.

## Summary

- Astrocytes play critical roles in maintaining a healthy brain environment. This is mediated through multiple homeostatic transporters, interactions with neurons and microglia, and functions at the blood brain barrier and synapses.
- Some astrocytes become ‘reactive’ in AD, while others show asthenia and loss of homeostatic functions. Astrocytes in AD reduce their support for synapses and show deficits in endolysosomal pathway components.
- There are increases in P2X_7_R mRNA and protein in AD, particularly in the vicinity of plaques. P2X_7_R activation in astrocytes influences synaptic activity and protein clearance pathways, and this may be one route by which P2X_7_R affects AD progression.
- Further exploration of the functional consequences of astrocytic P2X_7_R in AD may reveal novel cell-type specific targets for intervention.

## Abbreviations

A1R: adenosine A1 receptor
AD: Alzheimer’s disease
ADP: adenosine diphosphate
AMP: adenosine monophosphate
ApoE: apolipoprotein E
APP: amyloid precursor protein
AQP4: aquaporin-4
ATP: adenosine triphosphate
Aβ: β-amyloid
CNS: central nervous system
CSF: cerebrospinal fluid
EAAT (1/2): excitatory amino acid transporter (1/2)
FAK: focal adhesion kinase
GABA: γ amino-butyric acid
GABA-R: GABA receptor
GAT1/3: GABA transporter type 1/3
GFAP: glial fibrillary acidic protein
HSPB1: heat shock protein B1
HSPB25: heat shock protein B25
HSPG: heparin sulphate proteoglycan
LAMP1: lysosome associated membrane protein 1
LRP1: low density lipoprotein receptor-related protein 1
NFTs: neurofibrillary tangles
NMDAR: N-methyl-D-aspartate receptor
PET: positron electron tomography
PS1: presenilin 1
TFEB: transcription factor EB
TRPA1: transient receptor potential cation channel, subfamily A, member 1
UDP: uridine diphosphate
